# Community-Based Rehabilitation Post Hospital Discharge Interventions for Older Adults With Cognitive Impairment Following a Hip Fracture: A Systematic Review Protocol

**DOI:** 10.2196/resprot.3485

**Published:** 2014-09-16

**Authors:** Paula M van Wyk, Charlene H Chu, Jessica Babineau, Martine Puts, Dina Brooks, Marianne Saragosa, Katherine S McGilton

**Affiliations:** ^1^Department of KinesiologyFaculty of Human KineticsThe University of WindsorWindsor, ONCanada; ^2^Toronto Rehabilitation InstituteResearchUniversity Health NetworkToronto, ONCanada; ^3^Lawrence S BloombergFaculty of NursingUniversity of TorontoToronto, ONCanada; ^4^Toronto Rehabilitation InstituteLibrary and Information ServicesUniversity Health NetworkToronto, ONCanada; ^5^Faculty of Physical TherapyUniversity of TorontoToronto, ONCanada

**Keywords:** hip fracture, rehabilitation, cognitive impairment, community, at-home, intervention

## Abstract

**Background:**

Hip fractures among older adults remain a public concern. Consequences of a hip fracture include the subsequent decline in function and mobility for the older adult, and an increased burden placed upon their caregivers and the health care system. The consequences may be more challenging if an older adult also has a compromised cognitive reserve. Although rehabilitation programs have proven effective, the best practices and resources required to maintain the gains in function and mobility, to negate diminution of effect, and to enable this patient population to continue living at home are currently unknown.

**Objective:**

The objective of this study is to develop a systematic review protocol focused on identifying the evidence and evaluating the effectiveness of post discharge rehabilitation programs for older adults with a cognitive impairment following a hip fracture.

**Methods:**

The search strategy will include a combination of text words and subject headings relating to the concepts of cognitive impairment, dementia, delirium, cognitive reserve, and hip fractures. Searching various databases will identify peer-reviewed journal articles. There will be two independent reviewers who will screen the titles and abstracts to determine which articles comprise a rehabilitation intervention within a community setting prior to being included for a full article review. A data extraction form and an evidence and quality checklist will be used during the full article data analysis and synthesis. It is expected that there will be a paucity of studies that focus on post discharge rehabilitation interventions for older adults with cognitive impairment following a hip fracture, and few studies that use the same or similar outcome measures. However, if possible, a meta-analysis will be conducted on studies that used similar outcome methods.

**Results:**

This review will synthesize knowledge focusing on activities to maintain and restore function in older adult patients with cognitive impairment once they have completed their active rehabilitation program and return home. A synthesis of the findings will be conducted to determine which components of the interventions identified were the most advantageous to the patient population. The results will be used to develop a multi-faceted post discharge rehabilitation intervention aimed at enabling older adults to return and remain living at home after a hip fracture.

**Conclusions:**

The aim of this systematic review is to generate results that can be used to create interventions that focus on the care necessary to enable older adults to remain living at home post discharge from acute or inpatient rehabilitation care for a hip fracture. With the support and contributions by our associated knowledge users, this systematic review will be used to help inform procedures and policies to facilitate the necessary care and resources required by our patient population.

##  Introduction

### Older Adults and Hip Fractures

With approximately 35,000 a year in Canada and projections that indicate that the incidence will increase nearly four-fold by 2041, hip fractures remain a public health concern [1,2]. As a consequence, a hip fracture is often a catastrophic event that results in significant impairment in mobility, independence, and ability to live in the community [2]. The event of a hip fracture and the recovery period afterwards is complicated further by the presence of a cognitive impairment. Health care services are currently fragmented and limited, such that few patients with a cognitive impairment (CI), such as dementia or delirium, are able to access rehabilitation services [3-5]. Although there is evidence to suggest that community-based rehabilitation programs can be beneficial [6,7], there has yet to be a synthesis of studies that focused on community-based rehabilitation interventions post discharge from the hospital for older adults with a CI following a hip fracture.

In response to the need for better services for older adults with CI, previous research involved the development of a Patient-Care Rehabilitation Model for patients with a hip fracture including persons with CI (PCRM-CI) [8]. Outcomes at discharge revealed that patients in the PCRM-CI group were more likely to return home post discharge (vs being admitted to long-term care), despite having an average age above 80 years and multiple comorbidities. Longitudinal data showed that at 3 months post discharge mobility scores among persons with CI deteriorated significantly, but recovered to discharge levels at 6 months, thus, suggesting that the initial decline in mobility status may be mitigated if additional services are provided post discharge [8]. Nonetheless, if the progress made during inpatient rehabilitation is negated by discharging the patient home without proper resources or continued rehabilitation, then the health of the patient will be impacted, and the burden on caregivers and the health care system will be onerous. The next important step is developing interventions to reduce decline once the older person with CI is discharged home. A recent systematic review on early discharge planning and long-term outcomes for older adults, with and without CI post hip fracture, identified a gap in the knowledge of post discharge rehabilitation requirements that are necessary to achieve these recovery outcomes [9]. Thus, the review protocol presented in this paper will concentrate on community-based rehabilitation, post hospital discharge interventions, focused on older adults with CI following a hip fracture.

### Community-Based Rehabilitation Post Discharge Studies

Studies examining community-based rehabilitation post discharge from the hospital have found promising results. Binder et al [6] conducted a randomized controlled trial to determine if extended outpatient rehabilitation improved the physical function and reduced disability among older adults following a hip fracture. They compared progressive resistance training to low-intensity home-based exercise, and provided evidence in support of extended outpatient rehabilitation to improve physical performance, mobility, and quality of life, and reduce disability among community-dwelling older adults following a hip fracture. Home-based exercise programs can also result in an improved activity level compared to those who received usual care [6]. Orwig et al [7] defined usual care as the standard 2 to 4 weeks of inpatient rehabilitation, while home-based rehabilitation was described as a combination of aerobic activities, strength training, and self-efficacy based motivation [7]. Although the study demonstrated the feasibility of providing a home-based rehabilitation program, the study involved only women, primarily focused on exercise, and included only participants who were cognitively intact [7]. Shyu et al [10] implemented an interdisciplinary intervention that consisted of geriatric consultation, continuous rehabilitation, and discharge planning for Taiwanese older adults with CI following hospital discharge for a hip fracture [10]. In their 2 year follow-up study, it was found that the intervention improved the postoperative cognitive functioning [10]. Thus, there is some evidence to support that post discharge rehabilitation programs can have a positive impact on both the physical and cognitive functioning of older adults with CI following a hip fracture.

There have been several systematic reviews of interventions implemented to aid older adults with a musculoskeletal deficiency, such as hip fractures [11-19]. There are limitations, however, to these prior systematic reviews. In one review, Auais et al [19] found evidence indicating that extended exercise rehabilitation programs can have a significant impact on the functional abilities of individuals with hip fractures [19]. It was also determined that the community-based programs had larger effect sizes than home-based programs [19]. Mehta and Roy [16] made a comparison between rehabilitation outcomes from multiple modes of delivery (ie, home-based physiotherapy vs inpatient, outpatient, and no treatment) for patients following a hip fracture [16]. The results were inconclusive for indicating a mode of delivery with greater benefits to patients [16]. Stolee et al [17] compared the outcome of home-based versus inpatient rehabilitation interventions for older adults with musculoskeletal conditions [17]. The results suggested that the outcomes achieved by home-based rehabilitation programs were favorable in a number of studies [17].

There are pervasive limitations among prior systematic reviews. First, the results were not specific to patients with hip fractures, but covered a broad spectrum of musculoskeletal conditions [17]. Second, several reviews included only individuals who were cognitively intact [15,17,19]. Third, others examined only one rehabilitation modality (ie, exercise or physiotherapy) or one outcome variable [16,19]. Fourth, some studies only focused on inpatient interventions [11,18]. The systematic review protocol presented in this paper will look at the evidence for post discharge rehabilitation interventions in multiple modes in community settings, including home-based, for older adults with CI following a hip fracture. This systematic review will include studies that followed patients discharged from acute care and inpatient rehabilitation settings. *Rehabilitation* can encompass a vast number of activities, and can be broadly defined as health care activities that aim to improve patient well being and reduce caregiver burden [20]. Such activities may include, but are not limited to, exercise programs, strengthening, fall prevention, and home environment assessment and modifications. We are defining *community-based rehabilitation post discharge* as interventions initiated once an individual is discharged home after an acute phase of an illness is stabilized, and may include: (1) a physical component (eg, exercise, physiotherapy), (2) a cognitive component (eg, cueing, memory enhancing games), (3) a social activity or social engagement component, and (4) a pain management component. Post discharge rehabilitation takes place in the community in multiple settings, such as outpatient clinics or in the home of the individual. Key outcomes may include, function, mobility, quality of life, return to normal living, mortality rates, and engagement in the community. Thus, this review will examine a comprehensive range of outcomes including function, mobility, return to normal living, and activities of daily living, which has not previously been captured. The aim of this study is to systematically examine the literature for evidence that informs the design and implementation of the best discharge rehabilitation care pathway for older adults with CI following a hip fracture.

## Methods

### The Systematic Review

Following discharge from the hospital (ie, acute care or inpatient rehabilitation), the best care pathway will include interventions that: (1) maintain or continue to improve function, mobility, quality of life, and return to normal living; (2) prevent readmissions to the hospital or admissions to long-term care homes; and (3) reduce caregiver burden, health care costs, and mortality. This systematic review will look at the evidence for all modes of post discharge rehabilitation interventions in community settings, including home-based, for older adults with a CI following a hip fracture. Meta-analysis will be performed where possible. This systematic review will follow the methods described in the Cochrane Handbook for Systematic Reviews of Intervention, and be reported in compliance with the Preferred Reporting Items for Systematic Reviews and Meta-Analyses (PRISMA) guidelines [21].

### Data Sources and Search Methods

A comprehensive literature search will be conducted to identify any eligible studies. The search strategy will include a combination of text words and subject headings relating to the concepts of CI, dementia, delirium, cognitive reserve, and hip fractures (see Multimedia Appendix 1 for more information). Searching Medline, Medline In-Process, Pubmed, PsycINFO, Embase, CINAHL, A*MED, Ageline, The Cochrane Database of Systematic Reviews, Cochrane Central Register of Controlled Trials, Database of Abstracts of Reviews of Effect, and the Allied Health Evidence databases will identify peer-reviewed journal articles. An information specialist (JB) will conduct the database searches. Additionally, the search will include contacting the authors of included papers to request additional published or unpublished work, requesting unpublished work from experts in the field, and scanning conference abstracts and references from included studies.

### Article Screening and Selection

There will be two reviewers (CC, PMvW) that will independently screen the titles and abstracts of the articles. During the screening stage, the reviewers will determine whether the articles include: (1) a rehabilitation intervention, and (2) in the community interventions. The reviewers will use a screening form developed by the research team to guide them through the decision process for full-text screening.

A challenge that may arise is disagreement between independent reviewers when screening titles, abstracts, and full articles. In the case of a disagreement arising, all team members will be provided with the relevant material to come to a consensus decision. Research team meetings will be held on a regular basis to discuss articles, to ensure consensus in decisions, and to discuss any complications and findings.

### Inclusion Criteria

Publications that report the following will be considered for inclusion: (1) an intervention aimed at maintaining or improving function; (2) participants who are 65 years of age and older; (3) participants with CI; and (4) participants who suffered a hip fracture. Publication types to be included are randomized controlled trials, prospective (longitudinal), retrospective (longitudinal), cross-sectional, cohort, and quasi-experimental studies. According to the Cochrane Collaboration, a meta-analysis typically excludes nonrandomized controlled trials because of the higher risk of bias. However, we have chosen to include nonrandomized studies in order to collect a comprehensive overview of the evidence. To address concerns of bias, prior to the conduct of the meta-analysis, we will verify the risk of bias in each study, and based on these assessments, decide on the meta-analysis. The search will be limited to English and French articles. Each database will be searched for its entire scope of content, since the inception of the databases to the current date.

### Exclusion Criteria

Publications will be excluded if: (1) there is no community component to the rehabilitation program or intervention; (2) the study population includes patients with stroke, Parkinson’s, or frontal-temporal dementia, as these diseases have different physiological and behavioral markers; and (3) there is insufficient information in the publication to extract the necessary data (ie, editorials, expert opinions, qualitative studies, review articles, and publications that only include an abstract). Note; if there is insufficient information in the publication, where applicable, the authors will be contacted to retrieve additional information.

### Data Abstraction

For each of the included studies, the reviewers will independently extract data based on the developed extraction form. This includes information about the: (1) study (eg, aim of the study, sampling method, source of data, recruitment period, intervention duration, and study duration), (2) participants descriptors (eg, sample size, age, sex, type of hip fracture, type of CI, prefracture living location, and discharge location), (3) interventions (eg, components, setting, assessments, and scales used), (4) outcomes, (5) statistical analyses, and (6) randomization, allocation, and blinding methods.

### Data Analysis and Synthesis

It is expected that there will be a paucity of studies that focus on post discharge rehabilitation interventions for older adults with CI following a hip fracture, and few studies that use the same or similar outcome measures. If it is possible to conduct a meta-analysis of those studies which used similar outcome methods, the analysis of data will involve calculating a pooled effect size, calculating confidence intervals, testing for homogeneity, and determining publication bias following the methods as described by the Cochrane Collaboration using Review Manager software [22]. If it is determined that the outcome data is continuous in nature, a difference between means will be calculated.

Statistical heterogeneity will be determined using the i-squared and chi-square statistical tests [22]. If the i-squared value is equal to or less than 40 percent, a meta-analysis will be performed [22]. All statistically significant analyses will be interpreted using a cut-off *P* value of 5%. Funnel plots will be constructed to determine publication bias. Effect size will be on the x-axis, and sample size will be on the y-axis. Publication bias will be assumed minimal if the plot resembles a funnel with the base down.

As it is possible that the interventions may be implemented at different times or that assessments may be taken at different times, the included studies will be grouped into one of two categories. The first group will be categorized as “short-term”, in which the intervention or assessments take place within the first six months post discharge. If the intervention or assessments take place six months or later after the time of discharge, these studies will be categorized as “long-term”. If deemed appropriate, pooled estimates will then be created for both short-term and long-term studies for each outcome. To assess the impact of combining the data into these two groups, sensitivity analyses will be used.

CI may be defined within the studies as mild, moderate, or severe. Thus, a sub analysis will be conducted to determine if outcomes are more favorable among participants with less CI and vice versa. A sensitivity analysis will be performed by recalculating the meta-analysis to specifically make comparisons at each of the three levels of CI.

### Critical Appraisal Techniques

To evaluate the evidence and quality levels of the publications used in this review, we will use the Downs and Black checklist [23]. Many intervention studies in health care are not conducted as randomized controlled trials (RCTs); thus, it is imperative that we select a methodological quality assessment tool that can assess both RCTs and non-RCTs. The Downs and Black checklist was developed to assess both RCTs and non-RCTs, and has been previously used in a systematic review examining hip fracture rehabilitation practices of older adults [13,23]. The reviewers of the articles will also independently score the quality of the included studies, and any disagreements will be discussed and resolved by the research team.

## Results

This study aims to determine what, if any, rehabilitation interventions are provided to older adults with a CI who have experienced a hip fracture after they have been discharged from acute care or in-patient rehabilitation. Currently, a synthesis of the findings is being conducted to determine which components of the interventions identified were the most advantageous to the patient population. The projected completion date for the study is the end of 2014. The results will be used to develop a multi-faceted post discharge rehabilitation intervention aimed at enabling older adults to return and remain living at home after a hip fracture. Once the results of this review are known, the research team will organize an international symposium in Canada to present the findings to knowledge users and policy makers. We will invite influential individuals in the field to attend, including: (1) researchers, (2) policy makers, and (3) relevant networks and organizations, and health care professionals. The symposium will use the results of the review and the influence of the panel of decision-makers and experts to develop new guidelines for providing care for older adults with CI following a hip fracture to remain living at home safely.

##  Discussion

### Creating Interventions for Older Adults With Hip Fractures and Cognitive Impairment Post Discharge

For an older adult with a hip fracture, the subsequent decline in function and mobility can be debilitating, resulting in a great burden placed on their caregivers and the health care system. Thus, this review will synthesize knowledge focusing on activities to maintain and restore function in older adult patients with CI once they have completed their active rehabilitation program and return home. Our team of experts, including health care providers, rehabilitation practitioners, and researchers, has already been making great strides toward improving inpatient rehabilitation services received by older adults, especially those with CI [8]. The aim of this systematic review is to generate results that can be used to create interventions that focus on the care necessary to enable older adults to remain living at home post discharge from acute or inpatient rehabilitation care for a hip fracture. With the support and contributions by our associated knowledge users (physicians, policy makers, and family members of the patient population) this systematic review will be used to help inform procedures and policies to facilitate the necessary care and resources required by our patient population.

Ultimately, we expect our findings to benefit end users in countries around the world.

### Conclusions

The best practices and resources required to maintain the gains in function and mobility, to negate diminution of effect, and to enable this patient population to continue living at home are currently unknown. With the current initiative in Ontario focused on affording older adults the ability to “age in place” [24], it is imperative to understand the evidence that exists in the literature to enable older adults post discharge from hip fracture rehabilitation to remain living in their homes. Thus, the overall aim of this review will be to identify the evidence and evaluate the effectiveness of post discharge rehabilitation programs.

## Multimedia Appendix 1

Search strategy example for Medline Database: Ovid MEDLINE 1946 to January Week 2 2014.

## Abbreviations

CI: cognitive impairment
PCRM-CI: Patient-Care Rehabilitation Model of patients with a hip fracture including persons with CI
RCT: randomized controlled trial
