# Supplementary material for: Community-Based Rehabilitation Post Hospital Discharge Interventions for Older Adults With Cognitive Impairment Following a Hip Fracture: A Systematic Review Protocol
Source: JMIR Res Protoc. 2014 Sep 16;3(3):e47. doi: 10.2196/resprot.3485 (PMC4180327; doi:10.2196/resprot.3485)
Supplement: Supplementary file 1 [file resprot_v3i3e47_app1.pdf]

Multimedia Appendix 1

Search Strategy Example for Medline
Database: Ovid MEDLINE(R) 1946 to January Week 2 2014

Search Strategy:
1 exp dementia/ (114350)
2 delirium/ (5403)
3 wernicke encephalopathy/ (1353)
4 Delirium, Dementia, Amnestic, Cognitive Disorders/ (8485)
5 (dement* or alzheimer*).mp. (144174)
6 (lewy* adj2 bod*).mp. (5767)
7 ("organic brain disease*" or "organic brain syndrome*").mp. (972)
8 deliri*.mp. (18416)
9 (chronic adj2 cerebrovascular).tw. (411)
10 (cerebr* adj2 deteriorat*).tw. (168)
11 (pick* adj2 disease).mp. (2652)
12 (cerebral* adj2 insufficient*).tw. (56)
13 huntington*.mp. (12480)
14 binswanger*.mp. (524)
15 korsako*.mp. (1339)
16 (creutzfeldt or jcd or cjd).mp. (7279)
17 ((cognitiv* or cognition*) adj3 (impair* or disord*)).mp. (75678)
18 exp Cognition Disorders/ (58694)
19 or/1-18 (222034)
20 exp Hip Fractures/ (16928)
21 ((femur* or femoral*) adj3 (neck or proximal) adj4 fracture*).tw. (6750)
22 Hip/ (8533)
23 Fractures, Bone/ (45504)
24 22 and 23 (224)
25 ((hip* or pertrochant* or intertrochant* or trochanteric or subtrochanteric or extracapsular* or orthop*) adj4 fracture*).tw. (13927)
26 20 or 21 or 24 or 25 (23507)
27 19 and 26 (854)
